# Science, Innovation and Education as Pillars of High-Quality Implant Dentistry: Overcoming Challenges through Innovation Dictates Trends

**DOI:** 10.3390/jcm9051575

**Published:** 2020-05-22

**Authors:** Miguel de Araújo Nobre

**Affiliations:** 1 University Clinic of Stomatology, Faculty of Medicine, University of Lisbon, 1649-028 Lisbon, Portugal; mnobre@maloclinics.com; 2 Research and Development Department, Maló Clinic, 1600-042 Lisbon, Portugal

The evolution of implant dentistry since the discovery of the osseointegration concept has been remarkable. I was lucky enough to experience firsthand the early developments and later massification of the immediate loading and immediate function protocols through the All-on-4^®^ concept, since 1999, when Professor Paulo Maló informed me that after having consulted with Professor Bo Rangert, both agreed that a young clinician (me) should work on the study. Later in 2003, that young clinician was included as one of the three original co-authors in the first All-on-4^®^ concept manuscript [1]. I am very grateful to both these Professors for providing such opportunity and for mentoring me throughout the years. The development of immediate function protocols, at that time an innovation, was not an easy task, considering the resistance to change that usually accompanies any novelty. In this situation, the resistance to change was only broken through science, education, and a high dose of resilience from a hand-full of clinician-researchers, elevating immediate function and immediate loading protocols to the standard of care today.

Now, as then, it is the challenge of treating patients that drives innovation and dictates trends, always with high quality care as goal. The present Special Issue, “Implant Dentistry—Trends, Challenges and Innovations”, was assembled as a catalyst for innovation and a forum for presenting and discussing the treatment of challenging situations. Innovation and validation of concepts are displayed in this Special Issue, at different levels. Starting with immediate loading, a systematic review comprised of 34 prospective studies published in this Special Issue, estimated a mean weighted implant survival of 97.4%, together with stable peri-implant bone level changes, leading the authors to conclude that, under defined circumstances, it appears to have long-term predictability and a good success rate [2].

Currently, several concepts are being developed to increase the predictability of the rehabilitation, while aiming to reduce treatment time. One of the areas developed concerns the digital workflow [3], with much margin for improvement in the immediate future, either through an improved dental clinic—dental laboratory communication [4], or the potential application of customized prefabricated immediate provisional restorations [5]. According to a randomized controlled trial, no significant differences were registered in the success rate and marginal bone level between the immediate loading of dental implants employed from the digital workflow and the conventional implant treatment technique [6]. Still, considering the digital aspect of implant dentistry, computer-aided dynamic navigation for implant placement has emerged in recent years, allowing accurate implant placement and being considered as a safe and predictable procedure [7,8].

Implant dentistry was built on the challenges posed by the need to satisfactorily restore the patients’ dentition, with science standing as cornerstone. Evidence-based decisions should stand as foundations for the ultimate goal of providing high quality care to all [9,10]. This allowed developing protocols to successfully rehabilitate patients with challenging conditions, including patients with Hypodontia [11], or using zygomatic anchored implants as a successful alternative option for graftless restoration of the severely resorbed maxilla, including immediate loading protocols [12]. Moreover, innovation might be the response to overcome challenges that demand a skillful solution as registered in a retrospective study, where the use of a low-resorption collagen membrane coverage produced comparable results to suturing when attempting to seal ruptured Schneiderian membranes [13].

Innovation in implant dentistry includes methods, comprising both surgical [14] and prosthetic preparation techniques [15,16], post-operative interventions to increase implant stability [17], or new methods of measuring peri-implant bone [18], aiming to increase the probability of success in the long term. Innovation in implant dentistry also includes new materials and surface treatments. One such material is Polyetheretherketone (PEEK), considered a prime candidate to replace metallic implants and prostheses in orthopedic, spine and cranio-maxillofacial surgeries [19]. Additionally, substantial research efforts are undertaken in the development of bioactive implant surfaces, combining antimicrobial activity with osteogenic capacity to achieve correct osseointegration and long-term stability [20,21]. Innovation in implant dentistry is paramount, but only when the benefits are incremental or exponential when compared to the state-of-the-art. This makes it mandatory to perform comparisons between techniques in order to validate new methods, to otherwise account for reproducibility of current methods if no incremental gain is registered [22,23], or to evaluate the impact of previous methods in the long-term [24]. In any of the three scenarios, science emerges victorious.

The connection of a final implant-supported fixed prosthesis allowing high satisfaction of both patient and clinician stands as a landmark for success. However, it does not guarantee the maintenance of success in the long term. Peri-implant pathology (otherwise known as peri-implantitis, including all the anecdotal connections to imply a disease process similar to periodontitis—which is not!) [25,26] is regarded as the primary process for late implant failure. Therefore, it is important to develop tools that provide risk assessment based on science and data (rather than opinions), to enable both clinicians and patients to produce the necessary changes that increase the probability of success. The risk assessment tool for peri-implant pathology published in this Special Issue is the first in implant dentistry to be validated, registering an excellent discriminating capacity for indicating which patients were at greater risk during a five-year post-surgery follow-up period [27]. Moreover, this risk assessment tool was made open access through the Foundation for Oral Rehabilitation (https://www.for.org/en/treat/peri-implant-pathology-risk-assessment/take). Even when delivering high quality restorations and attempting to control potential risk factors, peri-implant pathology can occur. This poses a significant challenge for the recovery of the peri-implant complex, due to both the doubts concerning current treatments [28] and the early stage of the development of new treatments [29]. The first prognostic model for implants with peri-implant disease to be derived and validated in implant dentistry was published in this Special Issue, being useful to understand the prognosis of the implant(s) in question and shed light on the possible (favorable or unfavorable) outcomes [30].

Finally, there is education, whose absence would render translational science impossible or meaningless. A narrative review published in this Special Issue discusses the evolution of training and education in implant dentistry. The authors forecast a rapid evolution over the next decade, as technologies already being used in other industries (displayed and discussed in the manuscript) are incorporated into new and innovative learning models [31]. Furthermore, the merging of technological innovations is suggested to culminate in “Digital Dentistry”, which in turn will render “Digital Education of Digital Dentistry” inevitable.

It can be interpreted from the present Editorial (written as a summary of the 28 published manuscripts) that the present Special Issue provides an insightful reading to contemporary and future implant dentistry. The complete collection is provided open access for the reader to explore. For this to be possible, we must acknowledge the effort of all authors and reviewers that contributed for this Special Issue, whose commitment is deeply appreciated. Happy reading!
